# Let-7 Family microRNAs Regulate the Expression of the Urokinase-Receptor in Acute Myeloid Leukemia Cells

**DOI:** 10.3390/cells14090623

**Published:** 2025-04-22

**Authors:** Anna Li Santi, Mariaevelina Alfieri, Luigia Meo, Pia Ragno

**Affiliations:** 1 Department of Chemistry and Biology, University of Salerno, 84084 Fisciano (Salerno), Italy; 2 Clinical Pathology, Pausilipon Hospital, A.O.R.N Santobono-Pausilipon, 80129 Naples, Italy; m.alfieri@santobonopausilipon.it

**Keywords:** urokinase-receptor, uPAR, let-7 microRNAs, AML

## Abstract

The urokinase-receptor (uPAR) exerts multiple functions supporting most cancer hallmarks. Increased uPAR expression is associated with an unfavorable prognosis in several cancer types, including hematologic malignancies. We previously reported that three oncosuppressor microRNAs (miRNAs) can target the 3′untranslated region (3′UTR) of the uPAR mRNA and that uPAR mRNA is a competitive endogenous RNA (ceRNA) able to recruit oncosuppressor miRs, thus impairing their activity. We now show that uPAR mRNA can also be targeted by oncosuppressor members of the let-7 miRNA family in acute myeloid leukemia (AML) cell lines. Indeed, let-7a, let7d and let-7g directly target the 3′UTR of uPAR mRNA, thus down-regulating uPAR expression. These let-7 miRNAs are expressed in KG1 and U937 AML cells; their levels are high in KG1 cells, which express low uPAR levels, and low in the U937 cell line, expressing high levels of uPAR. Overexpression of these miRNAs down-regulates uPAR expression and impairs the adhesion to fibronectin and migration of U937 cells, without affecting their proliferation. Accordingly, the overexpression of specific inhibitors targeting these let-7 miRNAs efficiently increases uPAR expression in KG1 cells. These results indicate that selected let-7 miRNAs regulate uPAR expression and impair the adhesion and migration of AML cells.

## 1. Introduction

In recent decades, a huge number of scientific studies identified multiple types of non-coding RNAs, advancing the revolutionary concept that RNAs mostly serve to carry the information encoded in DNA and to translate it into protein [1]. In fact, non-coding RNAs constitute more than 90% of the RNAs and are involved in the regulation of most biological events [2]. ncRNAs are classified as small ncRNAs, such as micro-RNAs (miRNAs), and long ncRNAs (lncRNAs), depending on their length [3]. Among non-coding RNA species, miRNAs have been more extensively studied and biochemically and functionally characterized. MiRNAs are single-strand RNAs, typically composed of 20–23 nucleotides. miRNAs regulate gene expression by pairing with specific sequences usually located in the 3′ untranslated region (3′UTR) of target mRNAs, thus repressing translation [4]. miR expression is often dysregulated in cancer, thus causing increased expression of oncogenes or impaired expression of oncosuppressor genes [5].

We previously identified oncosuppressor miRNAs regulating the expression of the receptor of the urokinase-type plasminogen activator (uPAR) [6]. uPAR consists of a heavily glycosylated protein anchored by a glycosyl-phosphatidyl-inositol tail to the cell membrane [7]. uPAR regulates pericellular proteolysis by binding the uPA serine-protease, thus mediating cell migration through the extracellular matrix (ECM) [8]; uPAR is also able to bind vitronectin (VN), a provisional ECM component, particularly abundant in cancer tissues, thus mediating cell adhesion to ECM [9,10]. Further, uPAR can be associated with various cell-surface molecules, including integrins and receptors for formylated peptides (FPRs), which transduce uPAR-dependent signaling, thus promoting most activities dysregulated in malignant cells, as cell proliferation, survival, adhesion and migration [11,12]. In line with these multiple activities, uPAR expression is strongly increased in malignant cells; the up-regulation of uPAR levels is associated with worse prognosis, resistance to chemotherapy and reduced patient disease-free interval [13]. Interestingly, uPAR is also involved in hematopoietic stem cell mobilization into peripheral blood and homing and engraftment to BM, according to mechanisms potentially similar to those regulating the same processes of leukemia cells [14,15].

We previously reported that three oncosuppressor miRNAs, miR-146a, miR-335 and miR-622, can directly target uPAR mRNA in acute myeloid leukemia (AML) cells [6]. Among them, miR-146a is strongly involved in hematopoietic disorders and leukemia and miR-335 and miR-622 in childhood leukemias [16,17,18].

It is now well-established that the different species of intracellular coding and non-coding RNAs form a very complex network able to tightly control gene expression [2]. In fact, the impairment of this delicate balance can dysregulate the expression of genes crucial in various diseases, including malignant cells [5]. RNAs containing the same miRNA response elements (MREs), for instance, can bind the same miRNAs, thus competing with each other for the binding to them. These RNAs are indicated as competitive endogenous RNAs (ceRNAs). Indeed, the prevailing ceRNA acts as a miRNA sponge, recruiting specific miRNAs, thus reducing their availability in the cytoplasm and positively regulating the translation of their target [19].

Once it was demonstrated that uPAR mRNA is a target of oncosuppressor miRNAs, we reported its possible ceRNA activity in AML cells. In fact, we showed that the overexpression of uPAR 3′UTR in AML cells induces the increase of uPAR itself and of various pro-tumoral factors, including the oncogene c-Myc [20]. Further, we reported the expression of uPAR mRNA variants carrying the 3′UTR in AML cells and demonstrated that the more abundant variant, lacking exon 5, can act as a ceRNA, as well as the 3′UTR alone [21].

We now aim to identify new oncosuppressor miRNAs directly targeting uPAR mRNA, which could be potentially included in the complex RNA network driven by the uPAR 3′UTR.

**Figure 1:**
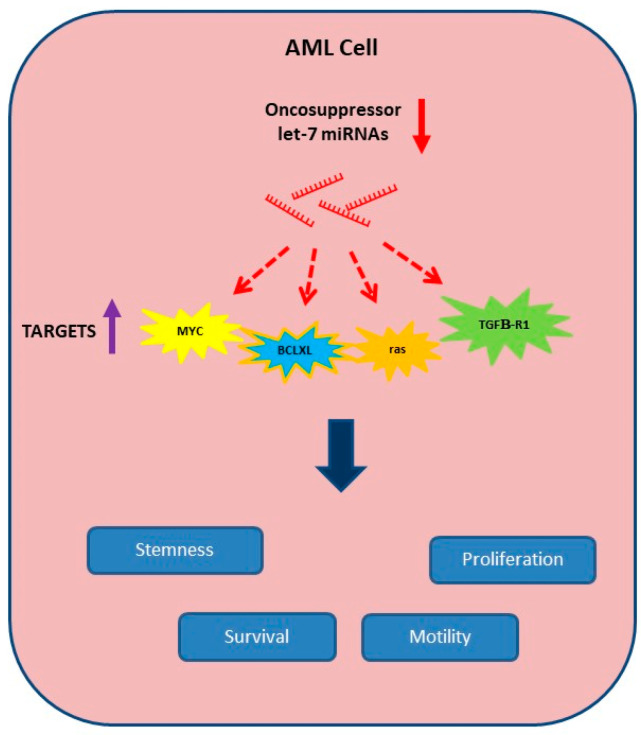
Let-7 miRNAs in AML. The down-regulated expression (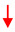) of oncosuppressor let-7 miRNAs in AML allows for the expression (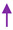) of their targets, thus promoting proliferation, survival, motility and stem-like phenotype.

We focused on the let-7 miRNA family. Let-7 was first identified after lin-14 in the nematode *Caenorhabditis elegans*; since then, various miRNAs have been isolated, and their number has been rapidly increased. The let-7 miRNA family is one of the most conserved miRNA families in the different animal species. In humans, there are 10 let-7 miRNAs encoded by multiple paralog genes. Let-7 miRNAs are not expressed in embryonic stem cells but start to be expressed later, remaining high in adult tissues; their expression is frequently dysregulated in cancer and influences the maintenance of cancer stem cells. Let-7 miRNAs usually function as tumor suppressors, contributing to impaired tumor growth and metastasis [22,23]. An important role of the let-7 family has also been proposed in the pathogenesis of hematological malignancies. The let-7 family is strongly involved in the biology of hematopoietic stem cells because it impairs the TGFβ pathway and promotes the Wnt pathway. The dysregulation of various let-7 miRNAs has been reported in AML; however, the trend of their dysregulated expression is often different between cytogenetically defined subgroups. The oncosuppressor activity associated with AML let-7 miRNAs depends on their capability to target oncogenes as c-Myc, Ras, BCLXL, HMGA, JAK, STAT3 and NIRF, regulating cell proliferation, apoptosis and motility (Figure 1); among the let-7 miRNAs dysregulated in AML, let-7b and let-7i have been reported to exert possible oncogenic roles in certain types of lymphomas [24].

On this basis, among the various let-7 miRNAs involved in AML, we focused on miRNAs showing oncosuppressor activity and decreased expression in newly diagnosed AML cases as compared with CD34^+^ cells from healthy controls, in particular let-7a, let-7d and let-7g [25,26]. We thus investigated whether uPAR expression can be regulated by these let-7 miRNAs, whose targets, in turn, could be indirectly regulated by uPAR 3′UTR.

## 2. Materials and Methods

### 2.1. Reagents

A pGL3 vector (containing a coding region for *Firefly* luciferase), pRLSV40 plasmid (containing a coding region for *Renilla* luciferase), dual-luciferase reporter assay system and CellTiter 96 AQueous One Solution Cell Proliferation Assay were purchased from Promega (Madison, WI, USA). Pre-miRs were obtained from Ambion (Austin, TX, USA) and Mercury LNA inhibitors from Exiqon (Vedbaek, Denmark). The TaqMan MicroRNA Assay was from Applied Biosystems (Foster City, CA, USA).

The anti-uPAR monoclonal antibody R4 (MON R-4-02), horseradish peroxidase-conjugated anti-mouse and anti-rabbit IgG were purchased from Thermo Fisher Scientific (Hanover Park, IL, USA). The protease inhibitor cocktail was from Sigma-Aldrich (Saint Louis, MO, USA). Anti-GAPDH antibodies (mAb G041 and rabbit polyclonal Y058203) were obtained from abm (Vancouver, BC, Canada).

The Nucleofector kit was from Lonza (Basel, Switzerland). The Protein Assay Dye Reagent Concentrate and iQ™SYBR Green Supermix were purchased from Bio-Rad (Hercules, CA, USA). The ECL detection kit and polyvinylidene fluoride (PVDF) membrane from Millipore (Burlington, MA, USA). The chemotaxis polyvinylpyrrolidonefree (PVPF) filters were from Whatman Int. (Kent, UK). QuantiTect Reverse Transcription kit, QIAquick PCR purification kit and QIAzol reagent were purchased from Qiagen (Hilden, Germany). Lipofectamine™ 2000, RNase-free DNAse I, the Superscript Reverse Transcriptase III, the Taq polymerase and the Platinum Superfi DNA polymerase were from Invitrogen (Carlsbad, CA, USA). The primers were produced by IDT (Coralville, IA, USA). The restriction enzymes were obtained from NEW ENGLAND BioLabs (Ipswich, MA, USA). Fibronectin was produced by Roche (Indianapolis, IN, USA).

### 2.2. Cell Culture

KG1 cells, derived from acute myelogenous leukemia, were cultured in Iscove’s Modified Dulbecco’s Medium (GIBCO, Thermo Fisher Scientific, Hanover Park, IL, USA) with a 20% heat-inactivated fetal bovine serum (FBS). U937 cells, derived from a promonocytic leukemia, were cultured in RPMI 1640 medium (GIBCO, Thermo Fisher Scientific, Hanover Park, IL, USA) with 10% FBS. Hela cells, derived from cervical carcinoma, were cultured in Dulbecco Modified Eagle Medium (GIBCO, Thermo Fisher Scientific, Hanover Park, IL, USA) with 10% FBS. Cell lines were from Sigma-Aldrich (Saint Louis, MO, USA).

### 2.3. In Vitro Transfection

Amaxa™ Nucleofector™ Technology (LONZA, Basel, Switzerland) was used to transfect by electroporation U937 and KG1 cells, following the manufacturer’s instructions. 20 pmol of miRNA precursors in 100 μL HBSS medium were used to transfect 1 × 10^6^ U937 cells whereas 50 nM LNA-ON inhibitors in 100 μL HBSS medium were used to transfect 2 × 10^6^ KG1 cells. In another set of experiments, 2 μg of plasmids pMS2-3′UTR or pMS2, together with 1 μg of pMS2BP-GST were co-transfected in 2 × 10^6^ KG1 cells in 100 μL HBSS medium (pMS2 and pMS2BP-GST plasmids were kindly provided from Dr. Myriam Gorospe). After electroporation, cells were diluted to 1.6 mL, plated in 35 mm plates and harvested at the indicated times.

Additionally, 2 × 10^5^ Hela cells were cultured in 35 mm plates for 16 h and then incubated for 5 h at 37 °C with 40 nM of selected miRNAs precursors and Lipofectamine^TM^ 2000 in serum-free medium; then, FBS was added up to 10% and cells harvested at different times.

### 2.4. Western Blot Analysis

Cells were lysed in 1% Triton X-100 added with protease inhibitors; proteins were quantified by a colorimetric protein assay. Specific bands were identified by 10% SDS-PAGE, transfer to a PVDF filter, blocking with 5% milk, hybridisation with primary antibodies and incubation with horseradish peroxidase-conjugated secondary antibodies and ECL.

### 2.5. Luciferase Assay

150 ng of the pGL3 plasmid, containing a 319 bp fragment encompassing uPAR 3′UTR (pGL3-3′UTR/uPAR) [6], 5 ng of pRLSV40 plasmid, containing the cDNA of *Renilla* luciferase for normalization, and 5 pmol of precursors of selected or control miRNAs were co-transfected by Lipofectamine^TM^ 2000 in HeLa cells previously cultured in 24-well plates (7 × 10^4^/well) for 24 h. Transfected cells were lysed after 24 h and the *Firefly* and *Renilla* luciferase activity was evaluated using the dual-luciferase reporter assay system, following the manufacturer’s instructions.

### 2.6. Real-Time RT-PCR Analysis

Total RNA was purified by lysing cells in QIAzol Reagent, according to the manufacturer’s instructions.

The levels of specific miRNAs were evaluated by the TaqMan MicroRNA Assay (Applied Biosystems, Foster City, CA, USA). 5 ng of total RNA was reverse transcribed; then, 3 μL of reverse transcription reaction was analyzed by quantitative PCR with a Bio-Rad IQ5 thermocycler (Bio-Rad, Hercules, CA, USA), following the manufacturer’s instructions. RNU6 was used as endogenous control.

To quantify uPAR-mRNA, 1 μg of total RNA was reverse transcribed using the superscript III reverse transcriptase KIT; 1 μL of a 1:10 dilution of reverse transcription reaction was analyzed by real-time PCR with a Bio-Rad IQ5 thermocycler, using iQ^TM^SYBR Green Supermix for qPCR kit. The levels of specific mRNAs were normalized to the internal glyceraldeyde-3-phosphate dehydrogenase (GAPDH) mRNA. The Primer3 software (https://primer3.ut.ee/) was employed to design the primers, used at 0.25 μM. The following primers were utilized: 5′-CTGGAGCTGGTGGAGAAAAG-3′ (forward primer) and 5′-CATGTCTGATGAGCCACAGG-3′ (reverse primer) for uPAR amplification; 5′-GAAGGTGAAGGTCGGAGTC-3′ (forward primer) and 5′-GA AGATGGTGATGGGATTTC-3′ (reverse primer) for GAPDH amplification. The formula 2^−ΔΔct^ was employed to calculate the relative level of expression.

### 2.7. MS2-Tagged RNA Affinity Purification

MS2-tagged RNA affinity purification (MS2-TRAP) indicates a methodology for the purification of ribonucleoprotein complexes that include RNA binding protein and/or ncRNAs bound to a specific mRNA [27]. MS2-TRAP was performed using plasmids pMS2 and pMS2BP-GST, kindly provided by Dr. Myriam Gorospe. The plasmid pMS2 has a pcDNA3 backbone containing 24 repeats of MS2 sequences and, upstream, a cloning site for inserting the cDNA prepared from the RNA of interest; the plasmid pMS2BP-GST (Glutathione SH transferase) codifies for a fusion protein, able to bind both MS2 sequences and glutathione-SH (GSH). In order to obtain the 3′UTR of uPAR to insert in pMS2, total RNA was isolated from U937 cells lysed in QIAzol. Total RNA was treated with RNase-free DNase I and reverse-transcribed using random hexamers and Superscript III. The 319 bp fragment encompassing uPAR-3′UTR was prepared by PCR using specific primers adapted to the HindIII/EcoRI sites (Hind III forward primer 5′-AAGCTTACCTGAAATCCCCCTCTCTC-3′ and ECORI reverse primer 5′- GAATTCTGGCCTTGTCCACTGGTACA-3′). The resulting fragment was inserted in the pMS2 vector in Hind III/EcoRI sites, in order to obtain the construct pMS2-3′UTR, encoding uPAR-3′UTR RNA tagged with 24 MS2 hairpins. The sequence of pMS2-3′UTR was assessed by sequence analysis (BMR Genomics, Padova, Italy). Then, 2 μg of pMS2 (control cells) or pMS2-3′UTR and 1 μg of pMS2BP-GST were co-transfected in 2 × 10^6^ KG1 cells using Amaxa™ Nucleofector™ Technology. After 48 h, cells were lysed in a solution containing 20 mM Tris-HCl at pH 7.5, 100 mM KCl, 5 mM MgCl_2_, 0.5% NP-40, protease inhibitors, RNaseOUT and 10 mM DTT. 2 mg of cell lysates were used for pull-down with GSH agarose beads. These beads, which bind with high-affinity GST, were washed and resuspended in ice-cold PBS 50:50. Lysates were incubated with GSH beads 3 h at 4 °C. Then, half of the beads were treated with DNase I and, subsequently, with proteinase K; RNA was extracted using acidic phenol. Real-time quantification of miRNAs present in the pulled-down RNAs was carried out by the TaqMan MicroRNA Assay. The remaining beads were treated with RIPA buffer (10 mM Tris-HCl, pH 7.4, 150 mM NaCl, 1% Nonidet P-40, 1 mM EDTA, 0.1% SDS and 1 mM DTT) and analyzed by Western blot with an anti-GST antibody to verify the pull-down efficiency.

### 2.8. Cell Adhesion Assay

1 μg of fibronectin (FN) or 1% heat-denatured BSA-PBS, as a negative control, were loaded onto flat-bottom wells of 96-well microtiter plates and incubated for 16 h at 4 °C. In order to block non-specific binding sites, coated wells were incubated with 1% heat-denatured BSA-PBS at room temperature for 1 h. 1.5 × 10^5^ U937 cells, transiently transfected for 24 h with indicated miRNAs, were loaded onto coated wells. Wells were washed after 2 h at 37 °C; attached cells were fixed with 3% paraformaldehyde at 37 °C in PBS, then with 20% methanol and, finally, stained with 0.5% crystal violet in 20% methanol. Staining was eluted by 0.1 M sodium citrate in 50% ethanol, pH 4.2, and the absorbance at 540 nm was measured with a spectrophotometer. The adherence to BSA was subtracted from the adherence to FN.

### 2.9. Cell Migration Assay

Cell migration was carried out in Boyden chambers with PVPF polycarbonate filters (5 µm pore size). 2 × 10^5^ U937 cells, transiently transfected for 24 h with indicated miRNAs, were loaded in the upper chamber in a serum-free medium; serum-free DMEM or 10% FBS-DMEM, as a chemoattractant, were added in the lower chamber. Cells were incubated for 2 h at 37 °C, 5% CO_2_; then, the cells on the lower surface of the filter were fixed in ethanol, stained with hematoxylin and counted at 20× magnification (10 random fields/filter).

### 2.10. Cell Proliferation Assay

Precursors of selected or control miRNAs were transiently transfected in U937 cells. 10 µL of cell suspension from each transfection was collected at 0, 24 and 48 h, diluted to 300 µL, distributed in three wells of 96-well plates and incubated with 20 µL/well of CellTiter 96 AQueous One Solution Reagent for 4 h at 37 °C, 5% CO_2_. Then, the absorbance was measured by an ELISA reader (Bio-Rad, Hercules, CA, USA).

### 2.11. Statistical Analysis

Differences between groups were evaluated by Student’s *t* test using PRISM software (GraphPad PRISM 2.01, San Diego, CA). *p* ≤ 0.05 was considered statistically significant.

## 3. Results

### 3.1. uPAR Is a Direct Target of Let-7 miRNAs in HeLa Cells

We investigated whether uPAR expression could be regulated by let-7 family miRNAs, focusing on let-7a, let-7d and let-7g miRNAs.

**Figure 2:**
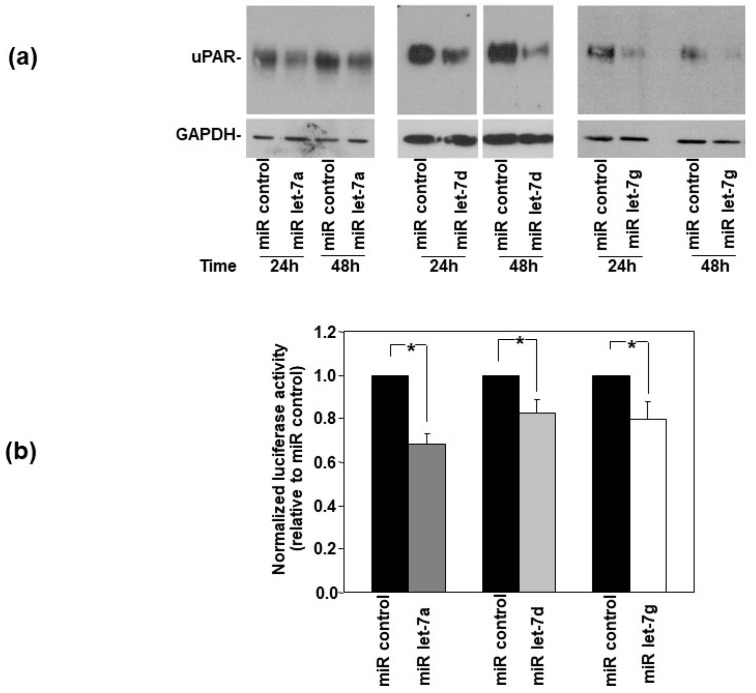
Let-7 microRNAs regulate uPAR expression in HeLa cells by targeting its 3′UTR. (**a**) The synthetic precursors of let-7a, let-7d and let-7g microRNA (miR) or of an off-target control miR were transfected in Hela cells; after 24 h and 48 h, cells were lysed and analyzed by Western blot analysis with a uPAR-specific antibody; filters were then re-probed with a mouse anti-GAPDH antibody for loading control. (**b**) Hela cells were transiently co-transfected with the pGL-uPAR3′UTR construct, containing the 3′Untranslated Region (3’UTR) of uPAR mRNA cloned immediately downstream of the stop codon of the *Firefly* luciferase and the pRLSV40 vector, containing the *Renilla* luciferase cDNA, and with the synthetic precursors of an off-target control miR or with the synthetic precursors of indicated miRs. The relative *Firefly* luciferase activity was assayed 24 h after transfection and normalized to the internal control *Renilla* luciferase. The columns represent the ratio between the normalized *Firefly* luciferase activity of cells transfected with let-7 miRs and the normalized *Firefly* luciferase activity of cells transfected with the control miR. Values are the mean ± SD of three experiments performed in triplicate. (*) *p* ≤ 0.05 as determined by Student’s *t* test.

Firstly, to investigate the ability of selected miRNAs to impair uPAR expression, their synthetic precursors were transfected in uPAR-expressing HeLa cervical cancer cells [8]. Western blot with an anti-uPAR antibody indicated that uPAR expression was reduced after both 24 h and 48 h of transfection with all transfected miRNAs, even if with a different efficiency (Figure 2a).

Then, in order to assess whether let-7 miRNAs down-regulated uPAR expression by directly targeting the 3′UTR of uPAR mRNA, the uPAR-3′UTR was inserted into the pGL3 vector immediately downstream of the *Firefly* luciferase reporter gene. Then, the pGL3-uPAR3′UTR construct and the pRLSV40 vector, containing the *Renilla* luciferase gene for normalization, were transiently co-transfected into Hela cells together with let-7a, let-7d or let-7g miRNA precursors or a control miRNA. After 24 h of transfection, the cells were lysed, and the activity of the luciferases was measured. All let-7 miRNAs significantly down-regulated the activity of the *Firefly luciferase* reporter gene, normalized to *Renilla* luciferase activity, as compared with the miRNA control (Figure 2b), demonstrating that those let-7 miRNAs can be recruited by uPAR-3′UTR, thus regulating the activity of the upstream reporter gene.

These results indicated that selected let-7 miRNAs regulate uPAR expression in HeLa cells by directly targeting its 3′UTR.

### 3.2. Let-7 miRNAs Are Differently Expressed in AML Cells

**Figure 3:**
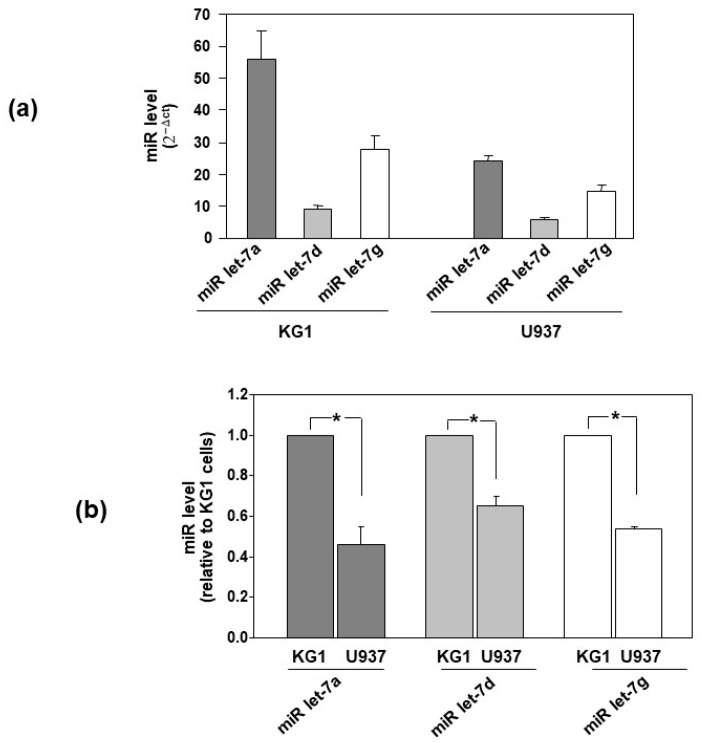
Let-7 miRNAs are differently expressed in AML cells. (**a**) The levels of indicated microRNAs (miRs) were analyzed by Taq-Man MicroRNA assay in KG1 and U937 cells and normalized to the internal control RNU6. The expression level was calculated with the formula 2^−ΔCT^. Values are the mean ± SEM of three experiments performed in triplicate; (*) *p* < 0.05 as determined by Student’s *t* test. (**b**) The same miR levels in U937 cells are expressed as fold changes relative to KG1 cells. The relative level of expression was calculated with the formula 2^−ΔΔCT^. Values are the mean ± SEM of three experiments performed in triplicate; (*) *p* < 0.05 as determined by Student’s *t* test.

Once it was demonstrated that selected let-7 miRNAs are potentially able to reduce the expression of uPAR, the levels of these miRNAs were investigated in two AML cell lines: KG1 (FAB M0/M1) cells, which express low levels of uPAR, and U937 (FAB M5) cells, which express high levels of uPAR [6]. The qRT-PCR analysis showed the expression of let-7 miRNAs in both AML cell lines (Figure 3a). miRNAs let-7a, let-7d and let-7g are more expressed in KG1 cells than in U937 cells (Figure 3b), consistently with the lower uPAR expression in KG1 cells than that in U937 cells. The observed levels of let7 miRs and uPAR expression in KG1 and U937 cells were consistent with the hypothesis that let-7 miRNAs may be involved in the regulation of uPAR expression in leukemia cells.

### 3.3. Let-7 miRNAs Regulate uPAR Expression in AML Cells

**Figure 4:**
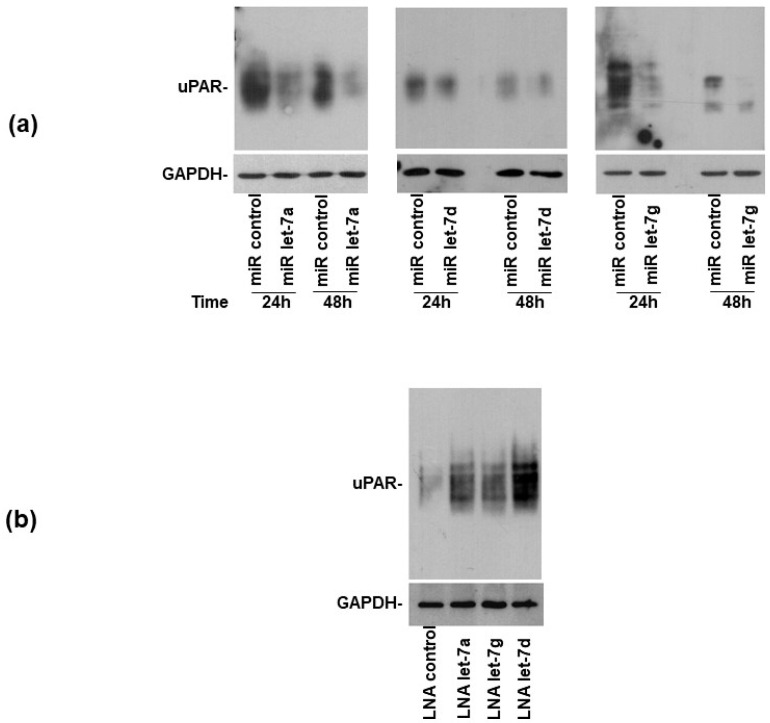
Let-7 microRNAs regulate uPAR expression in AML cells. (**a**) The synthetic precursors of let-7a, let-7d and let-7g microRNAs (miRs) or of an off-target control miR were transiently transfected in high-uPAR expressing U937 cells; after 24 h and 48 h, transfected cells were lysed and lysates were analyzed by Western blot with an anti-uPAR antibody; filters were then re-probed with a GAPDH-specific antibody for loading control. (**b**) LNAs specific for indicated let-7 miRs or a control LNA were transiently transfected in low-uPAR expressing KG1 cells; after 24 h, transfected cells were lysed and lysates were analyzed by Western blot with an anti-uPAR antibody; filters were then re-probed with a GAPDH-specific antibody for loading control.

Since the overexpression of miRNAs let-7a, let-7d and let-7g can control the uPAR expression in HeLa cells, we investigated whether they could also have the same effect in AML cells. Their synthetic precursors or a control miRNA were transiently transfected into U937 cells, expressing higher levels of uPAR than KG1 cells. 24 h and 48 h after transfection, cells were lysed and analyzed by Western blot with a uPAR-specific antibody. Western blot analysis showed that transfected let-7 miRNAs significantly reduced uPAR expression as compared with the control miRNA in U937 cells (Figure 4a). Thus, the overexpression of selected let-7 miRNAs can control uPAR expression in AML cells, as well as in HeLa cells.

miRNAs activity can be affected by locked nucleic acid (LNA)-oligonucleotides (ONs) [28]. To elucidate whether uPAR expression in AML cell lines could be regulated by endogenously expressed selected miRs, LNAs specific for selected let-7 miRNAs or a control LNA were transfected in KG1 cells, which express higher levels of let-7 miRNAs with respect to U937 cells, as shown in Figure 3. Western blot analysis showed that the transfection of LNA-ONs inhibiting endogenous let-7 miRNAs induced the increase in uPAR expression with respect to the transfection of control LNA-ON (Figure 4b).

**Figure 5:**
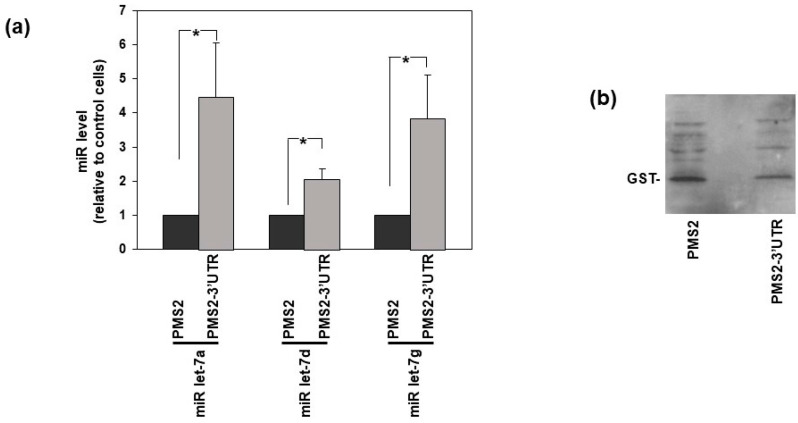
Let-7 microRNAs directly target uPAR 3′UTR in AML cells. KG1 cells were co-transfected with the plasmid pMS2 containing the 3′UTR of uPAR-mRNA (pMS2-3′UTR) or with the empty vector pMS2, and with pMS2BP-GST codifying a fusion protein, able to bind both MS2 sequences and glutathione-SH (GSH). Twenty-four hours later, transfected cells were lysed and incubated with GSH agarose beads. (**a**) The RNA pulled down by an aliquot of the GSH beads was extracted and the presence of indicated Let-7 miRs was shown by TaqMan MicroRNA Assay. Values are the mean ± SEM of three experiments performed in triplicate; (*) *p* < 0.05 as determined by Student’s *t* test. (**b**) The remaining beads were treated with RIPA buffer; eluted proteins were analyzed by Western blot with a GST-specific antibody to verify the pull-down efficiency.

We then used a different approach to further confirm that indicated endogenous miRNAs really bind the 3′UTR of uPAR mRNA, thus regulating its expression in AML cells. The MS2-tagged RNA affinity purification (MS2-TRAP) is a technique that allows to isolate miRNAs bound to their target RNA in the cell [27]. This method is based on the use of two plasmids: (i) the plasmid pMS2, consisting of a pcDNA3 backbone including 24 repeats of MS2 sequences and, upstream, a cloning site for inserting the cDNA of the potential target RNA; (ii) the plasmid pMS2BP-GST (MS2 binding protein-Glutathione SH transferase), that codify for a fusion protein, able to bind both MS2 sequences and glutathione-SH (GSH). The uPAR 3′UTR was inserted in the pMS2 vector, obtaining the construct pMS2-3′UTR, encoding uPAR-3′UTR RNA tagged with 24 MS2 hairpins. Then, KG1 cells, which express high amounts of miRNAs targeting uPAR mRNA, were co-transfected with pMS2 (control cells) or pMS2-3′UTR, and with pMS2BP-GST. The MS2BP should be able both to bind MS2-tagged RNA (in this case the uPAR 3′UTR, potentially bound by miRNAs) and to interact with the substrate of GST, the reduced glutatione (GSH). Thus, transfected cells were lysed and incubated with GSH agarose beads. The RNA pulled down by the GSH beads was extracted and the presence of let-7 miRNAs was shown by TaqMan MicroRNA Assay (Figure 5).

Altogether, these results suggest that let-7 miRNAs target the uPAR-3′UTR, thus contributing to the regulation of uPAR expression in AML cells.

### 3.4. Let-7 miRNAs Impair Adhesion and Migration of AML Cells

MiRNAs regulate most biological processes by regulating the expression of involved molecules [29]. Let-7 miRNAs down-regulate the expression of uPAR, which contributes to the control of cell proliferation, adhesion and migration mechanisms by associating with molecules expressed on the cell surface. Thus, the effects of let-7 miRNAs on these cell activities have been investigated.

The synthetic precursors of miRNAs let-7a, let-7d and let-7g or of a control miRNA were transfected in U937 cells; transfected cells were then tested in cell adhesion, migration and proliferation assays.

**Figure 6:**
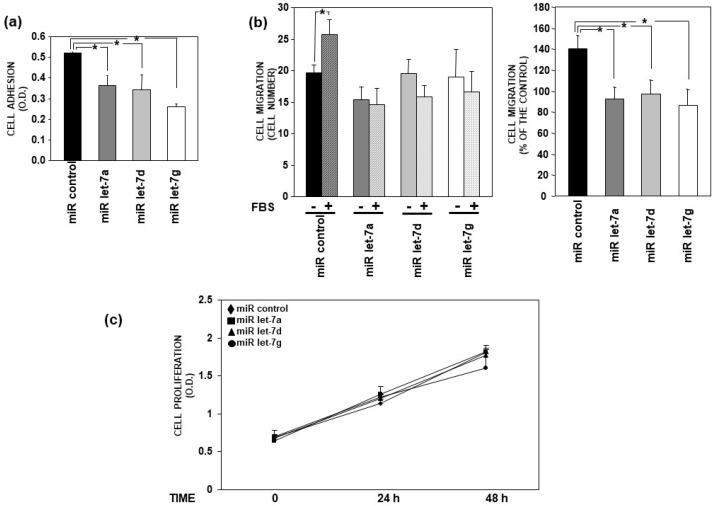
Let-7 microRNAs impair the adhesion and migration of AML cells. (**a**) The synthetic precursors of indicated let-7 microRNAs (miRs) or of a control miR were transiently transfected in U937 cells. Then, cells were incubated on fibronectin (FN)-coated wells (10 μg/mL) or BSA-coated wells (1%), as control. Adherent cells were fixed and stained; the staining was eluted, and its absorbance was measured by a spectrophotometer. The absorbance corresponding to control cells plated on BSA was subtracted from the absorbance corresponding to cells plated on FN. (**b**) The synthetic precursors of indicated let-7 miRs or of a control miR were transiently transfected in U937 cells. Migration towards 10% serum (FBS) of transfected cells was assessed in Boyden chambers. Migrated cells were stained with haematoxylin and counted (left panel). The percentage of cells that migrated towards FBS over the cells that migrated without chemoattractant was also evaluated (right panel). (**c**) The synthetic precursors of indicated let-7 miRs or of a control miR were transiently transfected in U937 cells. Then, at 0 h, 24 h and 48 h, 10 µL of cell suspension from each transfection was diluted to 300 µL, distributed in three wells of 96-well plates and incubated for 4 h with 20 μL of CellTiter 96 AQueous One Solution Reagent. The absorbance was then measured by an ELISA reader. (**a**–**c**) For each type of assay, values are the mean ± SEM of three experiments performed in quadruplicate; * *p* < 0.05, as determined by Student’s *t*-test.

Cell adhesion assays were performed on fibronectin (FN), a component particularly abundant in the extracellular matrix of bone marrow. U937 cells transfected with let-7 miRNAs adhered to FN significantly less than U937 cells transfected with the miRNA control (Figure 6a).

U937 cells transfected with the control miRNA efficiently migrated towards serum, used as a chemoattractant, in Boyden chambers; let-7 miRNAs overexpression significantly impaired U937 cell ability to migrate towards the same chemoattractant (Figure 6b).

In contrast, no significant effects on U937 cell proliferation were observed after the overexpression of let-7 miRNAs with respect to the control cells (Figure 6c).

Altogether, these results indicate that selected let-7 miRNAs negatively regulate the adhesion and migration of AML cells. This result may be due to let-7 miRNAs capability to affect uPAR expression, but the involvement of other molecules, whose expression can be down-regulated by these miRNAs, cannot be excluded.

## 4. Discussion

The increase in uPAR expression occurs in various tumors, including hematological malignancies, and is associated with unfavorable prognosis. In fact, uPAR is able to sustain most of the biological capabilities that tumor cells acquire during the multistep malignant development [13]. Thus, uPAR may represent an important tool in cancer diagnosis and prognosis and an interesting target in cancer therapeutics. Specific peptides, small molecules and monoclonal antibodies impairing uPAR interaction with its extracellular ligands, uPA and vitronectin, have been reported to affect tumor growth and metastatic processes [13,30].

uPAR was first identified in peripheral blood monocytes and in U937 AML cells [31]. uPAR expression, at protein level, was reported in monocytes, macrophages, neutrophils and myeloid precursors, as well as in myeloid malignancies, whereas hematopoietic stem cells were uPAR-negative [32]. Thereafter, uPAR expression in AML blasts was reported to be heterogenous but dependent on the FAB subtype, the highest expression being in the M4-M5 groups; furthermore, regardless of the FAB subtype, high uPAR levels correlated with a more aggressive phenotype of AML cells and with a worse prognosis [33,34]. uPAR expression at mRNA levels was confirmed in M4-M5 subtypes [35]. Increased levels of the soluble form of uPAR (suPAR) were reported in plasma from patients with AML, in particular in patients with residual disease after chemotherapy; suPAR levels correlated with the number of circulating tumor cells [36,37].

uPAR is implicated in the proliferation and survival of hematopoietic stem cells, in the mechanisms of their mobilization from and homing to the BM, mechanisms that may mirror those regulating the movements of malignant hematopoietic cells [14,15,38,39].

miRNAs contribute to the regulation of differentiation and activity of hematopoietic cells; their expression is often dysregulated in human malignancies [40,41].

We previously showed that uPAR mRNA may be a direct target of three oncosuppressor miRNAs in AML cells and blasts [6]; thus, uPAR mRNA may belong to the complicated cellular RNA network involved in the mechanisms regulating gene expression. In fact, uPAR mRNA may function as a competitive endogenous RNA (ceRNA) because its 3′UTR is able to recruit miRs in common with other mRNAs, thus allowing their translation; given that uPAR-recruited miRs are oncosuppressor miRs, the consequence is the expression of tumoral proteins [20]. Furthermore, we also identified uPAR mRNA variants carrying the 3′UTR and showed the ceRNA activity of one of these variants [21]. On this basis, we were interested in identifying new oncosuppressor miRNAs targeting uPAR, thus expanding and deepening the knowledge of the potential ability of uPAR to act as an oncogenic ceRNA.

We focused on let-7 family miRNAs, which play central roles as oncosuppressor miRNAs both in cancers and hematological malignancies [22,23,24,25,26].

A significant association of high let-7a expression with prolonged overall survival and event-free survival times was observed in cytogenetically normal AML patients [25]; its derepression, mediated by Lin28B inhibition, impairs cell growth, whereas its down-regulation leads to chemoresistance [42,43]. The expression of let-7d is down-regulated as compared with normal CD34+ cells in diagnosed AML patients [26]. A significant increase in let-7g expression was observed in leukemia cells associated with the longest latency of disease in AML model mice; the forced expression of let-7g induced the differentiation of leukemic blasts [44].

We now show that these miRNAs are expressed in KG1 and U937 AML cells at levels inversely related to uPAR expression, suggesting that uPAR expression may be regulated by these let-7 miRNAs. In fact, let-7 miRNAs can target uPAR 3′UTR, thus regulating the expression of the *Firefly* luciferase reporter gene. Furthermore, the overexpression of selected miRNAs reduces uPAR expression in U937 cells and, on the other hand, inhibitors of these let-7 miRNAs increase uPAR expression in KG1 cells, confirming their functional role in leukemia cell lines. Finally, we show that the overexpression of selected miRNAs affects the adhesion and migration of U937 cells, consistently with the role that uPAR plays in such cell activities. Interestingly, their overexpression does not exert any effect on cell proliferation, differently from the overexpression of previously identified uPAR-targeting oncosuppressor miRNAs [6].

Altogether, these results demonstrate that let-7 miRNAs regulate uPAR expression and are implicated in the regulatory mechanisms of cell adhesion and migration, probably also through the regulation of uPAR expression. On this basis, it can be hypothesized that, in turn, these oncosuppressor miRNAs might be recruited by uPAR mRNA, allowing the translation of their oncogenic targets. Thus, these results expand the group of pro-tumoral factors that can potentially be regulated by the ceRNA activity of uPAR transcripts, underlining the importance of down-regulating uPAR expression, besides blocking the protein activities, in therapeutic oncologic approaches.

## Data Availability

The original contributions presented in this study are included in the article. Further inquiries can be directed to the corresponding author(s).
